# “Blacklists” and “whitelists”: a salutary warning concerning the prevalence of racist language in discussions of predatory publishing

**DOI:** 10.5195/jmla.2018.490

**Published:** 2018-10-01

**Authors:** Frank Houghton, Sharon Houghton

**Affiliations:** Director, HEALR Research Group, Limerick Institute of Technology, Limerick, Ireland; Psychology Department, University of Limerick, Limerick, Ireland

## Abstract

This commentary addresses the widespread use of racist language in discussions concerning predatory publishing. Examples include terminology such as blacklists, whitelists, and black sheep. The use of such terms does not merely reflect a racist culture, but also serves to legitimize and perpetuate it.

The plague of racism is insidious, entering into our minds as smoothly and quietly and invisibly as floating airborne microbes enter into our bodies to find lifelong purchase in our bloodstreams. [1]

Maya Angelou

In 2008, Jeffrey Beall, a librarian at the University of Colorado in Denver, produced a list of potential, possible, or probable predatory journals and publishers [2]. Although Kirsten Bell has taken a more positive view of predatory publishing [3], considerable negative attention and concern has focused on the exponential growth of publishing ventures that prioritize profit over quality and engage in a litany of suspect and deceitful practices [4–10]. Further attention focused on the issue of predatory publishers through a number of highly publicized sting operations targeting such publishers [10–12].

The Western-biased [13], Eurocentric [14], and racist overtones of some aspects of the predatory publishing debate have already been noted [15]. However, in examining the emerging literature surrounding predatory publishing, it is striking how often the term “blacklist” is used to describe Beall’s list of potential, possible, or probable predatory journals and publishers [16–20]. Although Monica Berger discusses the terminology used in relation to predatory publishing, her examination fails to explore its racist aspect [21]. It is also notable that the term “blacklist” is often featured in quotation marks (as demonstrated here) [22], which appears to indicate that some authors are at least aware of the inappropriateness of such language. However, its use continues, and the new fee-for-access list recently developed by a private company (Cabell’s International) to replace Beall’s list is also routinely framed in this manner [23]. The use of this term is also apparent in respected academic magazines such as *University Affairs* [24], the *Times Higher Education Supplement* [25], and the *Chronicle of Higher Education* [26].

To compound the issue, it is also striking how often the term “whitelist” is used for a supposedly good, respectable, or safe list of publishers [20, 22, 27]. The racism in such “black is bad, white is good” metaphors is inappropriate and needs to cease. The black-white dualism explicit in these binary terms is often associated with Western thinking that is usually traced back to the work of Rene Descartes. Although the epistemological dualism of Descartes may be seen in earlier works by Plato and Aristotle, this way of thinking is often associated with the Enlightenment and the subsequent scientific revolution and industrial development [28–30]. Thus, a foundational ontological dualism accepted by many people in Western cultures includes the supposedly “natural” divides between subject-object, body-spirit, human-nature, and self-other. Such dualism extends into our conceptions of good-evil, sacred/divine-profane, and civilized-heathen/barbarian [31].

In this context, it is worth examining the origins of the term “blacklist” from the *Douglas Harper Etymology Dictionary,* which states that its origin and history is:

n.

also black-list, black list, “list of persons who have incurred suspicion,” 1610s, from black (adj.), here indicative of disgrace, censure, punishment (attested from 1590s, in black book) + list (n.). Specifically of employers’ list of workers considered troublesome (usually for union activity) is from 1888. As a verb, from 1718. Related: Blacklisted; blacklisting. [32]

It is notable that the first recorded use of the term occurs at the time of mass enslavement and forced deportation of Africans to work in European-held colonies in the Americas.

It is also interesting to observe that although the term “blacklist” is pervasive throughout the predatory publishing literature, equally racist terms such as “black sheep” [33, 34] and “black market” [35] are also frequently used in relation to predatory publishers. The term “black” in this context implies disreputable [36], shamed [37], illicit [36], or outcast [38].

Such terminology not only reflects racist culture, but also serves to reinforce, legitimize, and perpetuate it. On this issue, it instructive to read comments by Ossie Davis on the use of English as a racial affront:

the word WHITENESS has 134 synonyms; 44 of which are favorable and pleasing to contemplate…Only ten synonyms for WHITENESS appear to me have negative implications—and these only in the mildest sense…

The word BLACKNESS has 120 synonyms, 60 of which are distinctly unfavorable, and none of them even mildly positive…

Who speaks to me in my Mother Tongue damns me indeed!…the English Language…with which to survive at all I must continually be at war. [39]

Davis is not alone in his analysis of the legacy of racism in the use of the word “black” in the English language [40–42].

Despite the insubstantial protestations of some who would deny the connotations and impact of such language [43], the use of the terms “black” and “white” in the context of predatory publishing must be considered racist. It is important to evaluate the continued use of such racially charged terminology against the backdrop of the wider sociopolitical landscape and, notably, the emergence of populist racism and white supremacy on center stage of political life in the United States and elsewhere [44]. The centrality of racism and sexism in the 2016 US election campaign has been noted by many commentators [45]. The United States has experienced a growth of authoritarian populism based on the blatant use of racism, xenophobia, and Islamophobia by the Trump administration [46, 47]. Jerry Harris et al. address this issue by explicitly stating that:

At its core, his ruling power bloc consists of neo-liberal fundamentalists, the religious Right, and white nationalists. There are similarities between the new power bloc and fascism. [48]

It is important to remember that the medical literature is not immune to such influences and the growth of racism. The importance of language in racism and the use of coded racist terminology has been explored in-depth [49].

Evidence suggests the use of racially charged terminology such as “blacklist” includes librarians [19, 20, 27, 50]. It is imperative that such vocabulary ceases to be deemed acceptable. Examination of the history of terms such as “blacklist,” combined with the context of a growth in racist discourse, means that this is a real issue and not just a matter for idle academic debate. Finally, it is perhaps useful to conclude with a quotation by author N. K. Jemisin:

If the first words out of your mouth are to cry “political correctness!”,…chances are very, very high that you are in fact part of the problem. [51]
